# TCM Formula Xiaoyaosan Decoction Improves Depressive-Like Behaviors in Rats with Type 2 Diabetes

**DOI:** 10.1155/2015/415243

**Published:** 2015-10-05

**Authors:** Na Li, Qun Liu, Xiao-Juan Li, Xiao-Hui Bai, Yue-Yun Liu, Hong-Bo Zhao, Zhong-Ye Jin, Yu-Xia Jing, Zhi-Yi Yan, Jia-Xu Chen

**Affiliations:** School of Basic Medical Science, Beijing University of Chinese Medicine, Beijing 100029, China

## Abstract

The mechanism of depression with type 2 diabetes remains elusive, requiring further study. *Objective*. To evaluate the effect of TCM formula Xiaoyaosan on depressive-like behaviors in rats with type 2 diabetes. *Methods*. Rats were divided into 5 groups and drugs were administered during the model period of 21 days. The model of depressive-like behaviors in rats with type 2 diabetes was induced by a high fat diet, low doses of STZ injection, and chronic restraint stress for 21 days. The body weight, fasting blood glucose, ITT, OGTT, 5-HT, DA, depression behaviors, and morphological changes of formation were measured and observed. *Results*. After modeling, marked changes were found in model rats; behavioral analyses of rats indicated that this modeling method negatively impacts locomotor function. In the H&E staining, changes were found predominately in the CA1 and DG subregions of the hippocampus. After 21 days of treatment by fluoxetine and Xiaoyaosan, rats' body weights, behaviors and fasting blood glucose, and hippocampal formation were modified. *Conclusions*. A new model of depressive-like behaviors in rats with type 2 diabetes was successfully created. Xiaoyaosan and fluoxetine in this study independently contribute to exacerbate the disease progression.

## 1. Introduction

Depression in people with diabetes is globally prevalent. The condition affects quality of life [1], glucose control [2], nonadherence to treatment [3], cost of living [4], mortality, and life expectancy. According to the published studies, up to 45% of psychiatric disorders are not detected among the people suffering from diabetes. It has been identified that race, advanced age, and lack of health insurance were closely related to increased undertreatment for psychiatric disorders [5]. Chronically depressed individuals are more likely to have adverse effects associated with diabetes. One out of ten diabetes patients have been reported to suffer from apparent depressive symptoms, and as high as 25–30% of patients have inconspicuous depressive symptoms [6, 7]. It has been shown that people with depression and diabetes have higher mortality rates than individuals with diabetes exclusively, and the occurrence of self-harm and committing suicide is more frequent in patients with diabetes than in healthy population [8]. This indicates that the high prevalence of depression in diabetes patients needs further studies of the mechanism and medical treatment.

Xiaoyaosan decoction originated in* Taiping Huimin Heji Jufang*, created in the Song Dynasty of China (960–1127 AD). The decoction composition is composed of 8 crude herbs, Radix Angelicae Sinensis, Radix Paeoniae Alba, Poria, Radix Bupleuri, Radix Glycyrrhizae, Rhizoma Atractylodis Macrocephalae, Herba Menthae, and Rhizoma Zingiberis Recens. Xiaoyaosan decoction contains various chemical compounds, such as paeoniflorin, liquiritin, curcumin, and saikosaponins [9]. The decoction has been extensively used to treat mental diseases such as depression and the syndromes such as liver stagnation and spleen deficiency in traditional Chinese medicine clinical practice. Also, Xiaoyaosan decoction is used for the prevention and treatment of multiple-system diseases such as psychiatric disorders, neurological diseases, digestive system diseases, gynecologic diseases, and endocrine diseases [10–14]. Additionally, modified Xiaoyaosan has a significant effect to regulate diabetes [15].

While clinical studies provide evidence for the assessment of physiological context, an animal model is needed to provide the appropriate mechanistic characterization for a systemic pathology.

The objective of this research was to observe the effect of Xiaoyaosan decoction on depressive-like behaviors in rats with type 2 diabetes. Despite decades of research, little knowledge is recognized about the mechanisms supporting the depression combined with diabetes, which is quite complex. We expect our study in this paper will be able to provide empirical evidences with animal experiment.

## 2. Materials and Methods

### 2.1. Animal

170 ± 20 g male Wistar rats (SCXK(Jing)2012-0001) were housed under standard laboratory conditions 24 ± 1°C, 45 ± 15% relative humidity, and 12 h/12 h dark/light cycle with food and water freely available (SYXK(Jing)2011-0024).

### 2.2. Study Design

**Figure 1:**
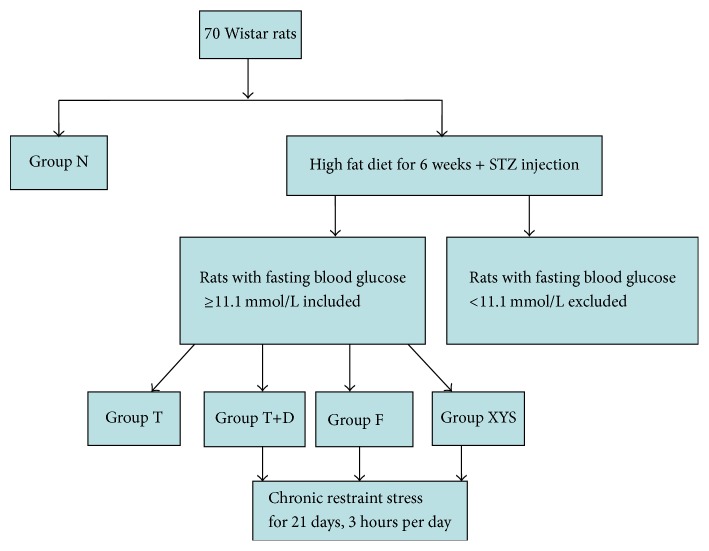
Study design.

Wistar rats were divided into two groups, 8 rats for normal group stressor (group N, no stressor plus deionized water), fed with standard diet ([SCXK(Jing)2009-0012]); 62 rats for the high fat diet group (compound feed: 68.8% of basic feed, 10% of sugar, 10% of lard oil, 10% of yolk powder, 1% of cholesterol, 0.2% of cholate [SCXK(Jing)2009-0008]). After six weeks of dietary treatment, rats in the high fat diet group were intraperitoneally injected with a single dose of streptozotocin (35 mg/kg; Sigma, USA). Fasting blood glucose was measured after three days and seven days. Forty-six rats with fasting blood glucose higher than 11.1 mmol/L were selected from the diabetes group. The other rats with fasting blood glucose lower than 11.1 mmol/L were excluded. The rats in the diabetes group were divided into four groups, including the type 2 diabetes group (group T, no stressor and deionized water), the depressive-like behaviors in rats with type 2 diabetes group (group T+D, stressor plus deionized water), the depressive-like behaviors in rats with type 2 diabetes model with fluoxetine group (group F, stressor plus fluoxetine), and the depressive-like behaviors in rats with type 2 diabetes model with Xiaoyaosan group (group XYS, stressor plus Xiaoyaosan), with 8 rats in each group. The model in group T+D was established on the basis of characteristics of emotional diseases and methods in the literature [16]. Some rats were randomly selected for chronic immobilization stress [17]. The rats were bound to a type T binding platform, which consists of the base (10 cm × 20 cm × 2.8 cm) and the upper part of the binding platform (22 cm × 6.6 cm). The front end had small frames for fixing the head and small grooves for limbs; the upper binding platform had two adjustable soft belts for, respectively, fixing the abdomen and chest. The rats were bound for 3 hours per day, randomly selected from 8 am to 7 pm to prevent the adaptation to a fixed binding time [18]. Study design see (Figure 1).

Experimental procedures were strictly in accordance with the Guide for the Care and Use of Laboratory Animals. The animal protocol was approved by the Committee on the Ethics of Animal Experiments of Beijing University of Chinese Medicine.

### 2.3. Preparation of Extracts of Xiaoyaosan Decoction

Xiaoyaosan decoction consists of 300 g of* Poria cocos* (Schw.) Wolf (*Poria*), 300 g of* Paeonia lactiflora* Pall. (Radix Paeoniae Alba), 150 g of* Glycyrrhiza uralensis* Fisch. (Radix Glycyrrhizae), 300 g of* Bupleurum chinense* DC. (Radix Bupleuri), 300 g of* Angelica sinensis* (Oliv.) Diels (Radix Angelicae Sinensis), 300 g of* Atractylodes macrocephala* Koidz. (Rhizoma Atractylodis Macrocephalae), 100 g of* Mentha haplocalyx* Briq. (Herba Menthae), and 100 g of* Zingiber officinale* Rosc. (Rhizoma Zingiberis Recens). These eight herbs were purchased from Beijing Tongrentang Co., Ltd. The 8 herbs were processed into dry extract in the Chinese medicine preparation room of the China-Japan Friendship Hospital (Beijing), following the Regulation on Processing of Traditional Chinese Medical Herbal Pieces of Beijing. All raw materials were extracted by boiling water three times, and then the decoction was dehydrated in vacuo (70°C) and ground into powder for use. The extraction rate of the dry extract was 18.8%, dosage of Xiaoyaosan = 6.17 × crud herbs ÷ 60 kg (normal human body weight) × extraction rate (actual dry powder/actual crude herbs). Xiaoyaosan dissolved in deionized water was gavaged at a dose of 3.854 g/Kg·d [19], one time per day, 1 mL/100 g bodyweight. 20 mg/capsule of fluoxetine dissolved in deionized water was gavaged based on body weight. Group N, group T, and group T+D were gavaged with deionized water.

### 2.4. Instruments and Method of High Performance Liquid Chromatography Coupled with LTQ Orbitrap Mass Spectrometry

1 g Xiaoyaosan powder was put into 25 mL of 70% methanol-water solution, then the mix solution was ultrasonic extracted for 30 minutes at room temperature, filtered at 0.22 *μ*m filter, stored at 4°C.

Accela High performance liquid chromatography and LTQ Orbitrap XL were purchased from Thermo Fisher Scientific Company (America); methanol (HPLC Grade) and formic acid (HPLC Grade) were purchased from Thermo Fisher Scientific Company (America); reference standards were purchased from the Chengmust Company, Sichuan Province, China.

Xiaoyaosan was performed on high performance liquid chromatography (HPLC) Accela 600 pump, LTQ Orbitrap XL (Thermo Fisher Scientific Company, America) using a SB-Aq column (4.6 × 250 mm, 5 micron, Agilent Technologies, USA), Capillary Voltage 2500 V–3000 V, Tubeleu 110 V, Scan range 100–1500, Sheath Gas 30 psi, and Aux Gas Flow 10 psi.

*Method*. The mobile phases comprised eluent A (0.1% formic acid) and eluent B (methanol). The gradient flow was as follows: 0~5 minutes, 30% B; 5~40 minutes, 30–90% B; 40~45 minutes, 90% to 100% B; 45~50 minutes, 100% B. The analysis was performed at a flow rate of 1.0 mL/min. The injection volume was 10 *μ*L.

### 2.5. Body Weight, Fasting Blood Glucose, Oral Glucose Tolerance (OGTT), and Insulin Tolerance Test (ITT)

Body weights were monitored and measured every 7 days.

Fasting blood glucose was measured via tail vein after overnight fasting using an ultrasensitive hand-held glucometer (Johnson and Johnson, USA).

For OGTT, rats were fasted overnight and gavaged with glucose (2.5 g/kg body weight). Glucose levels were measured both before and 30, 90, 120, and 180 minutes after glucose administration.

For ITT, rats were intraperitoneally injected with insulin (0.6 units/kg body weight), and blood glucose levels were measured both before and 30, 90, 120, and 180 minutes after insulin administration.

### 2.6. Open Field Test

The open field test was monitored by camera on day 21 of chronic stress. The activity of the rats was measured in a 100 cm × 100 cm × 40 cm cube with wood walls and wood floor, without ceiling (handmade), which is covered by black paint. The chambers are individually divided into 25 squares by yellow paint. The activity monitor camera was on the top of the middle square (Panasonic, Japan), and both horizontal and vertical movements were analyzed by Observer 5.0 software (Noldus, Netherlands) and EthoVision 3.0 software (Noldus, Netherlands). Each rat was placed in the central square and observed for 5 minutes, testing in the apparatus once. Scores were calculated by the amount of time, including the rat's movement speed, out zone definition (s), spent rearing (defined as standing upright on its hind legs), the number of crossings in the grid lines (it crossed with at least three paws), and licking frequencies.

### 2.7. Preparation of Serum

Venous blood samples were obtained after anesthetization from all the groups and collected in tubes and then centrifuged at 3000 r/min for 10 minutes at 4°C. Serum was collected after 4 hours and then quick-frozen in liquid nitrogen and stored in a −80°C freezer for measuring 5-hydroxytryptamine and dopamine.

### 2.8. 5-Hydroxytryptamine and Dopamine

5-Hydroxytryptamine (5-HT) (Lot: 20141130, 60087R) and dopamine (DA) (Lot: 20141130, 60088R) levels were measured by ELISA (Thermo Multiskan MK3, Finland) and the concentration was calculated according to the standard curves.

### 2.9. Hematoxylin and Eosin Staining (H&E Staining)

All rats were processed by deep anaesthesia with 10% chloral hydrate (0.4 mL/kg) and were transcardially perfused through the ascending aorta with 200–300 mL of cold saline, followed by 300 mL of 4% cold paraformaldehyde in 0.1 M phosphate buffer (PB, pH 7.4) for 25 minutes. The brain was taken out and postfixed in the same fixative at 4°C for 6–10 hours before immersing in a 20% sucrose solution in 0.1 M PB at 4°C. The brains were frozen in liquid nitrogen and sectioned (30 *μ*m) using a freezing microtome (Leica-CM 1900, Germany) at −20°C. Brain sections were then thawed and mounted onto microscope slides that were previously coated with 10% polylysine. The slides were stored in a freezer (−70°C) prior to use [20].

H&E staining was performed as follows: after dewaxing with xylene, sections were stained with hematoxylin (Sigma, America) and eosin solution (Sigma, America).

### 2.10. Image Analysis

Image analysis was completed by the use of an image analyzer (MIAS99) with a color video camera (JVC TK-C1381) and an Olympus BX50 microscope.

### 2.11. Statistical Analysis

All numerical data were expressed as mean ± standard deviation ($\overset{-}{x}\pm s$). A mixed design analysis of variance (ANOVA) by SPSS 17.0 for Windows was used to analyze significant differences, with *P* < 0.05 considered significant.

## 3. Results

### 3.1. Compositional Analysis of Xiaoyaosan by HPLC-LTQ-Orbitrap-MS

**Figure 2:**
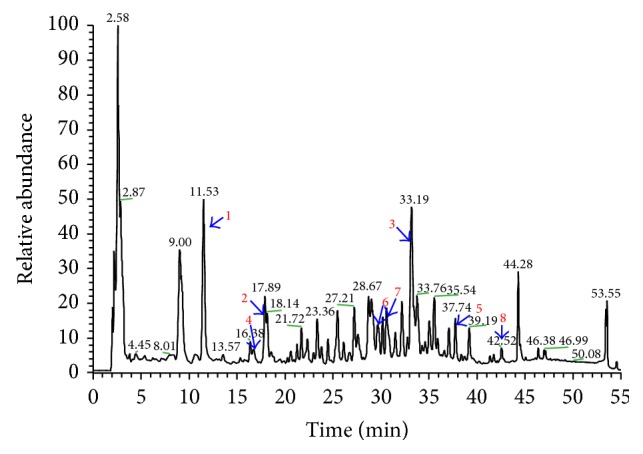
HPLC-LTQ-Orbitrap-MS chromatogram of eight ingredients from Xiaoyaosan samples. In Figures 2 and 3, HPLC-LTQ-Orbitrap-MS chromatogram of eight matched references in Xiaoyaosan sample from Tongrentangs 1, 2, 3, 4, 5, 6, 7, and 8 represents paeoniflorin, liquiritin, glycyrrhizic acid, ferulic acid, saikosaponins A and C, curcumin, and* Poria cocos* alcohol.

**Figure 3:**
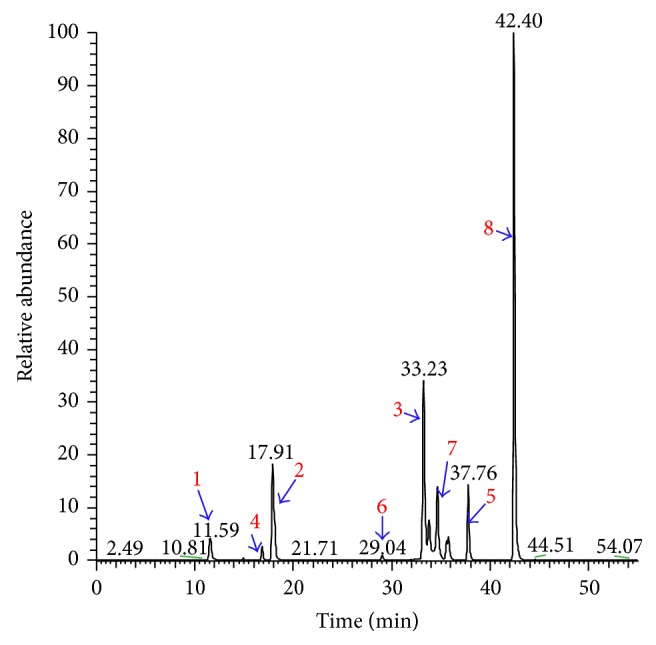
HPLC-LTQ-Orbitrap-MS chromatogram of eight reference standards. In Figures 2 and 3, HPLC-LTQ-Orbitrap-MS chromatogram of eight matched references in Xiaoyaosan sample from Tongrentang 1, 2, 3, 4, 5, 6, 7, and 8 represents paeoniflorin, liquiritin, glycyrrhizic acid, ferulic acid, saikosaponins A and C, curcumin, and* Poria cocos* alcohol.

Eight compounds, including paeoniflorin, liquiritin, glycyrrhizic acid, ferulic acid, saikosaponins A and C, curcumin, and* Poria cocos* alcohol in Xiaoyaosan samples were determined by HPLC-LTQ-Orbitrap-MS. The eight compounds are active ingredients in Radix Paeoniae Alba, liquorice,* Angelica sinensis*, Radix Bupleuri, fresh ginger, and* Poria cocos*, respectively. The alignment of the compounds with extracts of Xiaoyaosan indicated that eight compounds could match the corresponding peaks of Xiaoyaosan by the same HPLC-LTQ-Orbitrap-MS eluted system (Figures 2 and 3). The results suggested that the eight compounds might be quality control references of Xiaoyaosan.

### 3.2. Body Weight at Days 0, 7, 14, and 21

Compared with group N, rats in the other groups showed a significant decrease in the body weight at days 0, 7, 14, and 21 (*P* < 0.05, *P* < 0.01). Rats in group XYS showed a significant increase in body weight at days 14 and 21 compared with group T+D (*P* < 0.05), but there was no significant difference between group F and group T+D (*P* > 0.05) (Figure 6).

### 3.3. Fasting Blood Glucose

Compared with group N, rats in the other groups showed a significant increase in the fasting blood glucose at days 0, 7, 14, and 21 (*P* < 0.01). Rats in group XYS and group F showed a significant decrease in the fasting blood glucose at day 21 compared with group T+D (*P* < 0.05) (Figure 7).

### 3.4. Insulin Tolerance Test (ITT)

Compared with group N, rats in other groups showed a significant increase in the fasting blood glucose at 0, 30, 60, 120, and 180 minutes (*P* < 0.01), and it did not return to normal at 180 minutes. Compared with group T+D, there were significant differences in group XYS and group F at 30, 60, 120, and 180 minutes (*P* < 0.05, *P* < 0.01) (Figure 8).

### 3.5. Oral Glucose Tolerance Test (OGTT)

Compared with group N, rats in other groups showed a significant increase in the fasting blood glucose at 0, 30, 60, 120, and 180 minutes (*P* < 0.01), and it did not return to normal at 180 minutes. Compared with group T+D, there were significant differences in group XYS and group F at 30, 120, and 180 minutes (*P* < 0.05, *P* < 0.01), but the blood glucose did not recover to normal after 180 minutes (Figure 9).

### 3.6. 5-Hydroxytryptamine (5-HT) and Dopamine (DA) in Serum

Compared with group N, rats in the other groups showed no significant change in 5-HT and DA (*P* > 0.05). Rats in group T showed a significant increase in 5-HT compared with group T+D (*P* < 0.05). Rats in group XYS showed a significant increase in DA compared with group T+D (*P* < 0.05) (Figures 10(a) and 10(b)).

### 3.7. Scores on Open Field Activity

**Figure 4:**
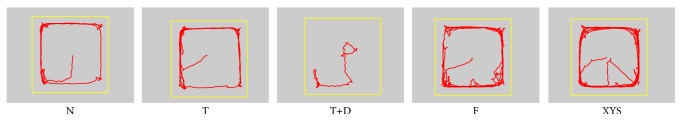
Representative behavior track plot reports of rats in different groups as assessed by open field test using video tracking software, indicating that this modeling method negatively impacts locomotor function. In group XYS and group F, rats showed improvement in locomotor function.

Open field tests were conducted at day 21 of the chronic restraint stress (Figures 11(a), 11(b), 11(c), 11(d), 11(e), and 4). In these behavioral tests, there were significant differences (*P* < 0.01, *P* < 0.05) among the rats in group N and group T+D, group T+D and group XYS, and group T+D and group F, with no differences between group N and group XYS, indicating that the model of depressive-like behaviors in rats with type 2 diabetes was successfully established, and the behavior of rats in group XYS and group F resumed to normal levels after 21 days treatment of Xiaoyaosan or fluoxetine in spite of the exposure to chronic restraint stress.

### 3.8. H&E Staining

**Figure 5:**
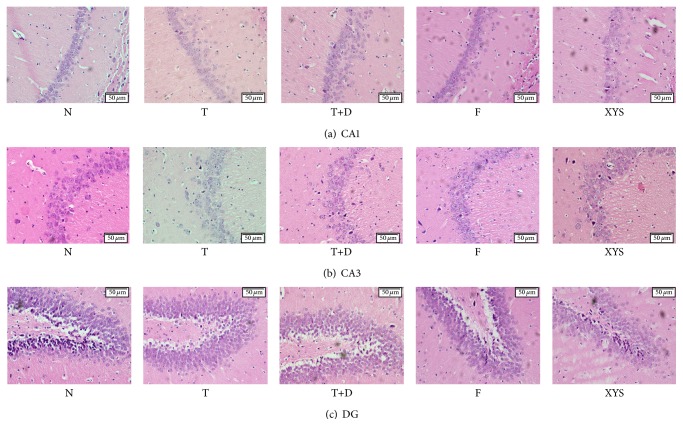
Histology of rat hippocampal structure in CA1, CA3, and DG subregions. Changes were found predominately in the CA1 and dentate gyms (DG) region of the hippocampus, rather than the CA3 subregion as seen in the pictures. Compared with group N and group T, obvious damage in hippocampal neurons appeared condensed and pyknotic in group T+D after high fat diet, STZ injection, and chronic restraint stress. The chromatins were in order, neurons increased, and less condensed and damaged neurons were found in group XYS and group F, compared with group T+D.

**Figure 6:**
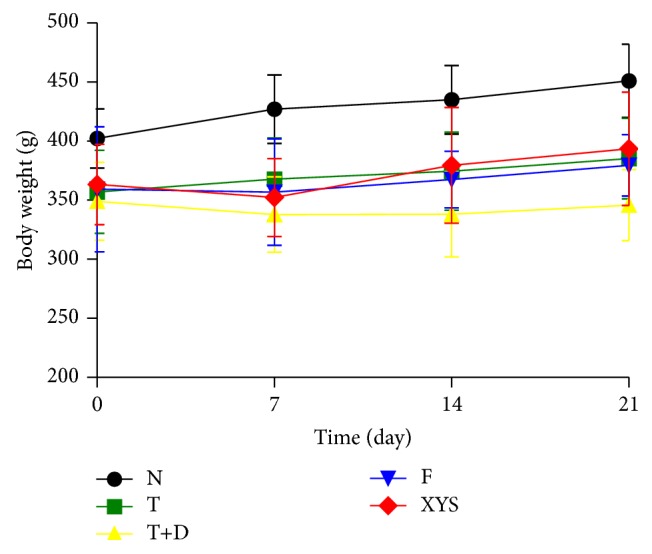
Body weight (g) of the rats in each group. Data are expressed as $\overset{-}{x}\pm s$, ^#^ *P* < 0.05, and ^##^ *P* < 0.01 versus group N; ^∗^ *P* < 0.05, ^∗∗^ *P* < 0.01 versus group T+D.

**Figure 7:**
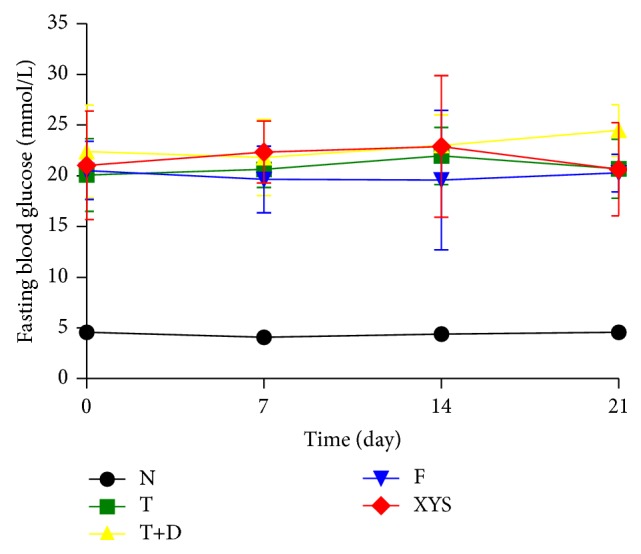
Fasting blood glucose (mmol/L) of rats in each group. Data are expressed as $\overset{-}{x}\pm s$, ^#^ *P* < 0.05, and ^##^ *P* < 0.01 versus group N; ^∗^ *P* < 0.05, ^∗∗^ *P* < 0.01 versus group T+D.

**Figure 8:**
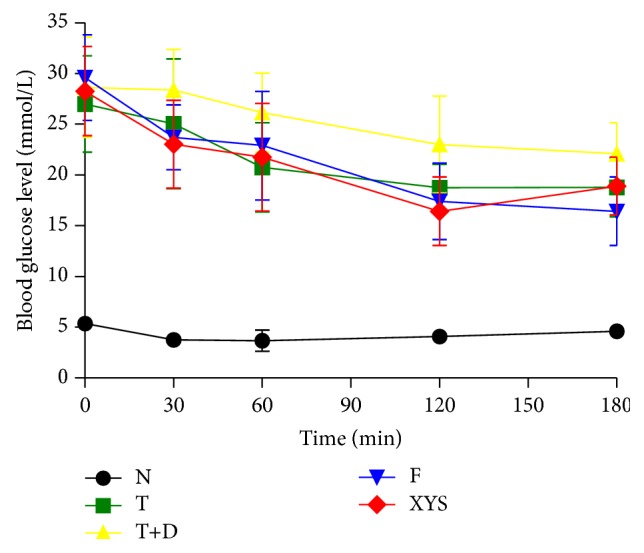
ITT (mmol/L) of rats in each group. Data are expressed as $\overset{-}{x}\pm s$, ^#^ *P* < 0.05, and ^##^ *P* < 0.01 versus group N; ^∗^ *P* < 0.05, ^∗∗^ *P* < 0.01 versus group T+D.

**Figure 9:**
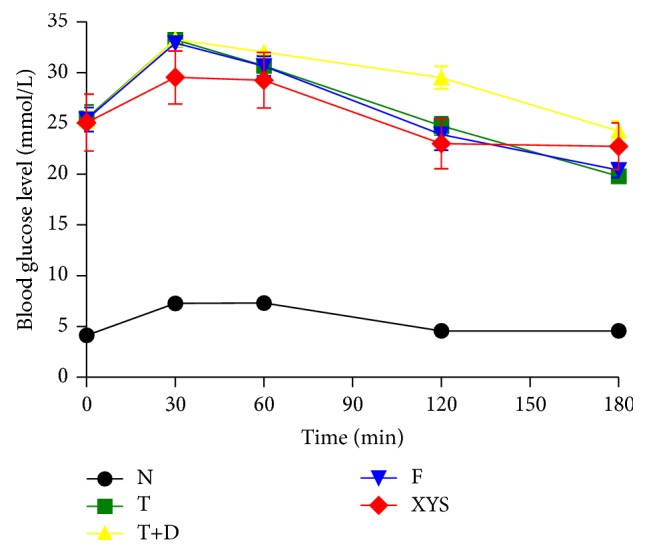
OGTT (mmol/L) of rats in each group. Data are expressed as $\overset{-}{x}\pm s$, ^#^ *P* < 0.05, and ^##^ *P* < 0.01 versus group N; ^∗^ *P* < 0.05, ^∗∗^ *P* < 0.01 versus group T+D.

**Figure 10:**
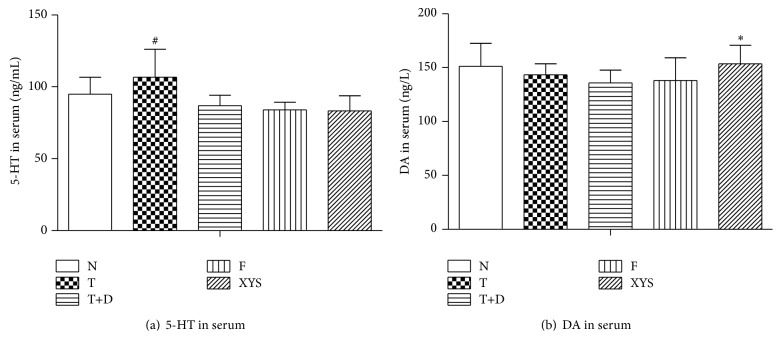
5-Hydroxytryptamine (5-HT) and dopamine (DA): (a) 5-HT (ng/mL) of rats in each group. (b) DA (ng/L) of rats in each group. Data are expressed as $\overset{-}{x}\pm s$, ^#^ *P* < 0.05 versus group N; ^∗^ *P* < 0.05 versus group T+D.

**Figure 11:**
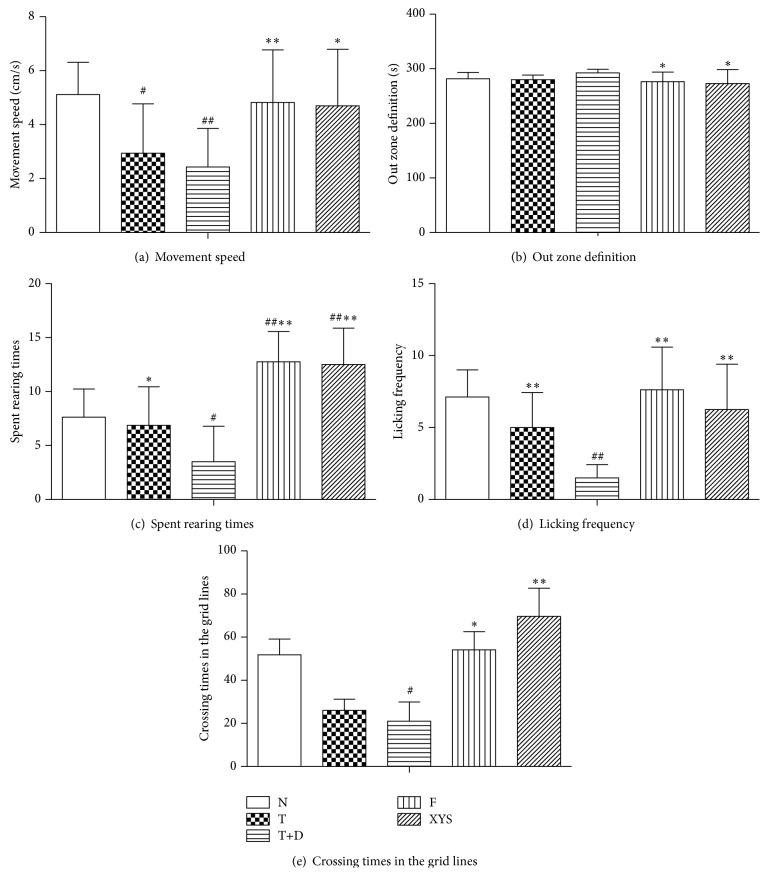
Scores on open field activity. (a) Movement speed (cm/s) in 5 minutes of open field test. (b) Out zone definition (s) in 5 minutes of open field test. (c) Spent rearing times in the grid lines times in 5 minutes of open field test. (d) Licking frequency in the grid lines times in 5 minutes of open field test. (e) Crossing in the grid lines times in 5 minutes of open field test. Data are expressed as $\overset{-}{x}\pm s$, ^#^ *P* < 0.05, and ^##^ *P* < 0.01 versus group N; ^∗^ *P* < 0.05, ^∗∗^ *P* < 0.01 versus group T+D.

Paraffin embedded brain sections were dewaxed and stained with hematoxylin and counterstained with eosin. The hippocampal structure was observed by electron microscope. Changes were found predominately in the CA1 and dentate gyms (DG) region of the hippocampus, rather than the CA3 subregion as seen in the pictures. The effect of chronic restraint stress on the histology of rat hippocampal structure (CA1, CA3, and DG) showed that the formation of the rat model in the rat's hippocampus induced neuronal loss or death and impacted locomotor function. Compared with group N and group T, obvious damage in hippocampal neurons in other groups appeared condensed and pyknotic. It indicated that marked neuronal degeneration, the death of CA1 pyramidal neurons in the hippocampus, is consistent with stress-related psychiatric illnesses [21]. Compared with group T+D, the chromatins were in order, neurons increased, and less condensed and damaged neurons were found in group XYS and group F. Compared with group T+D, there were signs of recovery in group F and group XYS after treatment, indicating that Xiaoyaosan and fluoxetine had an effect on model rats (Figure 5(a) CA1, Figure 5(b) CA3, and Figure 5(c) DG).

## 4. Discussion

As a traditional Chinese formula consisting of multiple compounds, Xiaoyaosan targets both depression and diabetes. The constituent chemical compounds, such as paeoniflorin, liquiritin, curcumin, and saikosaponins A and C, are active ingredients as antidepressants [22–26], while curcumin and paeoniflorins have antidiabetic effects [27–30]. The HPLC-LTQ-Orbitrap-MS chromatogram results showed that the eight compounds, including paeoniflorin, liquiritin, glycyrrhizic acid, ferulic acid, saikosaponins A and C, curcumin, and* Poria cocos* alcohol derived from Radix Paeoniae Alba, liquorice,* Angelica sinensis*, Radix Bupleuri, fresh ginger, and* Poria cocos*, respectively, could match corresponding peaks of reference standards by the same HPLC-LTQ-Orbitrap-MS eluted system. The results suggested that the 8 compounds might be quality control references of Xiaoyaosan. As the components are complicated, the analytical work and the evaluation of the disassembled prescription in detail should be further studied. The work is still ongoing.

In this study, after a high fat diet intake and STZ injection, the model rats' sugar tolerance was impaired. A significant body weight decrease has been observed after 21 days of chronic restraint stress. The neurotransmitter 5-hydroxytryptamine and dopamine, which play a role in central fatigue [31], mood regulation, movement, learning, and memory including energy metabolism and diet intake [32], were changed. Moreover, behavioral studies that use open field test to measure locomotor activity and depression [33] showed a progressive decline in the exploration of environment and a negative impact on spontaneous locomotor activity, which also contribute to depression. The hippocampus is especially vulnerable to stress-induced damage [34] and plays a role in executive function and working memory, involving in extinction of learning [35]. In the H&E staining, changes were found predominately in the CA1 and DG subregions of the hippocampus, instead of the CA3 subregion as shown in the pictures. It is unclear why changes are revealed in CA1 and DG subregions of the hippocampus rather than in the CA3 subregion. It might be that CA1 pyramidal neurons and DG granule neurons have a greater propensity to facilitate induced condensed, pyknotic, and damaged neurons than do CA3 pyramidal neurons. These results are consistent with those in the previous works reporting that patients with depressive disorders are usually accompanied by changes in the hippocampus [36]. Therefore, in this study, a new model of depressive-like behaviors in rats with type 2 diabetes was successfully established to study depression with type 2 diabetes disease. This is more humane and milder than the chronic unpredictable stress, which may provide the information applicable for further human clinical research.

In the clinical trial, fluoxetine was reported to improve insulin-mediated glucose disposal in obese patients, without changes in body weight [37], or improves glycaemic control in elderly type 2 diabetic patients [38]. It was also reported that the utility of stress management training could establish long-term glycemic control in type 2 diabetes [20]. In our previous study, Xiaoyaosan had almost the same effect on depression in rodent animals. Results from our HPLC-LTQ-Orbitrap-MS confirmed that Xiaoyaosan consists of antidepressive and antidiabetic components such as paeoniflorin, liquiritin, glycyrrhizic acid, ferulic acid, saikosaponins A and C, curcumin, and* Poria cocos* alcohol. We therefore created the hypothesis indicating that Xiaoyaosan is able to downregulate the blood glucose in depressive-like behaviors in rats with type 2 diabetes.

After 21 days of treatment, the body weight and blood glucose in Xiaoyaosan and fluoxetine treatment groups changed, and dopamine increased. Furthermore, rats also showed the improvement in locomotor function, which is corresponding to the previous studies regarding the Xiaoyaosan decoction modifying impairment of cellular plasticity and improving mood disorders by exerting neuroprotective and neurotrophic properties [20]. With neuroprotective properties, Xiaoyaosan decoction may ameliorate the impairment of neuron injury in the morphological observation. At the end of chronic restraint stress, these behavioral indicators were back to baseline when the model rats were treated with Xiaoyaosan or fluoxetine. Based on behavior research and morphological observation, it proved that Xiaoyaosan can produce significant antidepressant-like effect. Despite these findings, the blood glucose did not return to normal levels after the treatment, but the blood glucose showed a decline after treatment that is consistent with the hypothesis. The mechanism underlying the pathophysiology of mood disorders and type 2 diabetes is still unclear, so more investigations at the molecular level will be provided in future studies.

## 5. Conclusion

The mechanism of depression with type 2 diabetes remains elusive. The depressive-like behaviors in rats with type 2 diabetes provide an animal model in this study, which is a tool for investigation of the mechanism. The key point of this study is to demonstrate that Xiaoyaosan has a potential antidepressive and antidiabetic effect on depressive-like behaviors in rats with type 2 diabetes. The results confirmed and extended previous findings, implying that Xiaoyaosan in this study independently contributes to exacerbating disease state or disease progression. Furthermore, these results are consistent with the results in previous reports that Xiaoyaosan plays an important role in the treatment of neurodegenerative diseases, helps to maintain neuronal survival, and enhances rehabilitation and regeneration of neurons after injury. In summary, this study provides new evidence for the clinical application of the Xiaoyaosan decoction. However, further studies at molecular level are still needed to elucidate the mechanism for Xiaoyaosan in the treatment of depressive-like behaviors with type 2 diabetes, which will provide information for further evaluation in clinical trial.
